# Supplementary material for: Deletion of wfs1 Impairs Oligodendrocyte Precursor Cells Dorsal Distribution and Myelination Through the wfs1-hmgcs1 Axis in Zebrafish
Source: Biology (Basel). 2026 Aug 16;15(16):1402. doi: 10.3390/biology15161402 (PMC13510151; doi:10.3390/biology15161402)
Supplement: Supplementary file 1 [file biology-15-01402-s001.zip › File S1-Transcriptome sequencing data and differentially expressed genes (DEGs) in wfs1-- zebrafish at 5 dpf/2.KEGG/wfs1KOvsWT/DOWN/src/dre04621.html]

dre04621


- dre:323944

- Down regulated genes

323944(-4.98347167377177)

- dre:368481

- Down regulated genes

368481(-2.13889418398421)

- dre:403131

- Down regulated genes

403131(-4.42862381698358)

- dre:557239

- Down regulated genes

557239(-2.67898858596673)

- dre:436718

- Down regulated genes

436718\_1(-6.85571323128554)

- dre:100334992

- Down regulated genes

100334992(-1.17993350985631)
- dre:100149641

- Down regulated genes

100149641(-2.12200822650433)

- dre:373107

- Down regulated genes

373107\_1(-7.07514976036473)

- dre:436734

- Down regulated genes

436734\_1(-7.3292737184465)

- dre:100000242

- Down regulated genes

100000242(-6.34512572021214)

- dre:564295

- Down regulated genes

564295\_2(-1.27307321576804)

- dre:561021

- Down regulated genes

561021\_1(-4.68369619674599)

- dre:100002946

- Down regulated genes

100002946(-1.40391573648291)

Close
